# Efficacy of inhaled HYdrogen on neurological outcome following BRain Ischemia During post-cardiac arrest care (HYBRID II trial): study protocol for a randomized controlled trial

**DOI:** 10.1186/s13063-017-2246-3

**Published:** 2017-10-23

**Authors:** Tomoyoshi Tamura, Kei Hayashida, Motoaki Sano, Shuko Onuki, Masaru Suzuki

**Affiliations:** 1 Department of Emergency and Critical Care Medicine, Keio University School of Medicine, 35 Shinanomachi, Shinkuku-ku, Tokyo, 160-8582 Japan; 2 The Center for Molecular Hydrogen Medicine, Keio University, 2-15-45 Mita, Minato-ku, Tokyo, 108-8345 Japan; 3 Department of Cardiology, Keio University School of Medicine, 35 Shinanomachi, Shinkuku-ku, Tokyo, 160-8582 Japan

**Keywords:** Out-of-hospital cardiac arrest, post-cardiac arrest syndrome, hydrogen gas inhalation

## Abstract

**Background:**

Hydrogen gas inhalation (HI) improved survival and neurological outcomes in an animal model of post-cardiac arrest syndrome (PCAS). The feasibility and safety of HI for patients with PCAS was confirmed in a pilot study. The objective of this study is to evaluate the efficacy of HI for patients with PCAS.

**Methods/design:**

The efficacy of inhaled HYdrogen on neurological outcome following BRain Ischemia During post-cardiac arrest care (HYBRID II) trial is an investigator-initiated, randomized, double-blind, placebo-controlled trial designed to enroll 360 adult comatose (Glasgow Coma Scale score < 8) patients who will be resuscitated following an out-of-hospital cardiac arrest of a presumed cardiac cause. The patients will be randomized (1:1) to either the HI or control group. Patients in the HI group will inhale 2% hydrogen with 24% to 50% oxygen, and those in the control group will inhale 24% to 50% oxygen for 18 h after admission via mechanical ventilation. Multidisciplinary post-arrest care, including targeted temperature management (TTM) between 33 °C and 36 °C, will be provided in accordance with the latest guidelines. The primary outcome of interest is the 90-day neurological outcome, as evaluated using the Cerebral Performance Categories scale (CPC). The secondary outcomes of interest are the 90-day survival rate and other neurological outcomes. This study will provide 80% power to detect a 15% change in the proportion of patients with good neurological outcomes (CPCs of 1 and 2), from 50% to 65%, with an overall significance level of 0.05.

**Discussion:**

The first multicenter randomized trial is underway to confirm the efficacy of HI on neurological outcomes in comatose out-of-hospital cardiac arrest survivors. Our study has the potential to address HI as an appealing and innovative therapeutic strategy for PCAS in combination with TTM.

**Trials registration:**

University Hospital Medical Information Network (UMIN), 000019820. Registered on 17 November 2015.

**Electronic supplementary material:**

The online version of this article (doi:10.1186/s13063-017-2246-3) contains supplementary material, which is available to authorized users.

## Background

Anoxic neurological injury is a significant source of morbidity and mortality in cardiac arrest (CA) survivors [1]. Currently, targeted temperature management (TTM) is the only treatment that has both laboratory and clinical supportive data and is used to improve outcomes in patients with post-cardiac arrest syndrome (PCAS) [2, 3]. Although the optimum protocol for TTM has yet to be established, maintaining the body temperature between 32 °C and 36 °C, rather than not treating the fever, is now implemented as the gold standard for improving outcomes in patients with PCAS [4, 5]. Several drugs that block toxic metabolite production have shown promise in mitigating anoxic neurological injuries in animal PCAS models. However, these drugs have not been demonstrated to improve outcomes in clinical trials [6, 7]. Thus, a medical breakthrough is warranted in post-CA care, and we are in pursuit of a novel and simple therapeutic approach.

The unique antioxidative and antiapoptotic properties, as well as the potential therapeutic applications, of molecular hydrogen (H_2_) were first reported in 2007 [8]. Since then, the efficacy of H_2_ has been extensively studied in various animal models and preliminary clinical studies. In addition to its radical-scavenging effect, H_2_ has been reported to regulate gene expression and various signal transduction pathways by modifying the free radical chain reaction-dependent generation of oxidized phospholipid mediators [9]. Although the specific mechanisms underlying the pleiotropic effects demonstrated by H_2_ in various animal models have not been fully elucidated, the clinical translation of H_2_ is now attracting positive attention [10]. Improved survival rates, better neurological outcomes, and attenuated histological damage have been reported with hydrogen gas inhalation (HI) in a PCAS rat model. The salutary effect of H_2_ was comparable to that of TTM [11, 12]. With the unique features of H_2_, specifically its pleiotropic effects, high permeability, convenient administration, and lack of obvious adverse effects, HI has the potential to improve survival and neurological outcomes in patients with PCAS. Recently, we reported that HI is a feasible and safe approach for selected comatose post-CA patients [13]. Therefore, in this trial, we will evaluate whether HI is effective, compared with conventional oxygen inhalation, in improving 90-day neurological outcomes in comatose patients resuscitated after out-of-hospital cardiac arrest (OHCA) of a presumed cardiac cause.

## Methods/design

### Study design

The Efficacy of inhaled HYdrogen on neurological outcome following BRain Ischemia During post-cardiac arrest care (HYBRID II) trial is an investigator-initiated, randomized, placebo-controlled, double-blind multicenter superiority trial (RCT) with two parallel groups. Patients will be allocated 1:1 to either the intervention group or the control group. This RCT will be conducted at approximately 15 institutions in Japan. The goal of this trial is to evaluate the efficacy of HI on neurological outcomes in comatose patients who are resuscitated following OHCA of a presumed cardiac cause. This trial is registered with the University Hospital Medical Information Network (UMIN) clinical trials registry (UMIN000019820). A completed Standard Protocol items: Recommendations for Interventional Trials (SPIRIT) Checklist is available in Additional file 1.

### Inclusion criteria

The inclusion criteria are as follows: patients aged between 20 and 80 years; OHCA of a presumed cardiac cause with return of spontaneous circulation (ROSC) in a prehospital setting or in the emergency room (ER); unconscious (Glasgow Coma Scale [GCS] score < 8) after ROSC; systolic blood pressure (≥80 mmHg) with or without fluid loading, vasopressors, and/or inotropes; written informed consent obtained from the patient’s next of kin; and < 6 h lapsed after ROSC before HI initiation.

### Exclusion criteria

The exclusion criteria are as follows: known prearrest Cerebral Performance Categories scale (CPC) 3 or 4, known limitations in therapy and “do not resuscitate” order, association with trauma, intracranial bleeding, acute stroke, acute aortic dissection, malignancy in terminal stage, pregnancy, acute intoxication, oxygen (O_2_) saturation < 94% with 50% O_2_ inhalation and adequate positive end-expiratory pressure (PEEP), cardiopulmonary bypass use, and determined to be inappropriate for the study by the study investigators.

### Enrollment and withdrawal

The study participants are unconscious and hence are unable to provide consent themselves. Therefore, before a subject’s participation in the trial and after a full explanation of the study’s purpose and procedures, written informed consent will be obtained by site investigators from the patient’s next of kin present at the hospital. The participants will be asked whether they wish to continue participating in the study after they regain competent consciousness. The patient will be withdrawn from the study if the patient or the patient’s next of kin withdraws consent. All data obtained from that patient will be discarded. Patients will be enrolled by investigators at each hospital.

### Randomization, blinding, and allocation

Owing to the limitations in transporting large gas cylinders in a timely manner and storing them in limited spaces at facilities, pairwise transportation and randomization will be performed. Thus, cylinders will be sequentially numbered from 1 to 360, and each of the 180 pairs (e.g., 1 and 2, 3 and 4,…) will be transported simultaneously from a storage center to a facility upon request. One of each pair of gas cylinders will contain hydrogen gas, and the other will contain a control gas. Because two cylinders will be used per patient, each pairwise shipment will contain four cylinders. Which of the gas cylinders of each pair contains hydrogen or control gas will be determined by selecting a random binary number generated by the Bernoulli distribution (probability 0.5) using a computer, and this information will be entered into an allocation table. The allocation table will be kept secret by the assignment manager until the time of the key opening. Before a pair of gas cylinders is transported from a storage center, the gas cylinders will be covered by cylindrical covers so that the contents of the cylinders (hydrogen or control gas) cannot be recognized. Every set of two patients at each center will be randomly allocated 1:1 to the hydrogen and control groups while maintaining blindedness.

### Study intervention

**Fig. 1:**
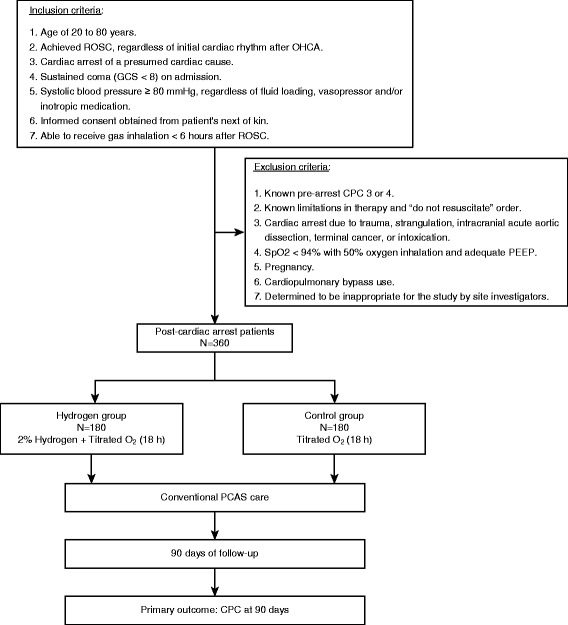
Flow diagram of the study design. Patients will be randomly allocated 1:1 to the hydrogen group or the control group using content-blinded cylinders. Trial gas inhalation will be initiated after intensive care unit admission and continued for 18 h. Multidisciplinary treatments, including targeted temperature management, will be performed in all patients according to the latest International Liaison Committee on Resuscitation (ILCOR) guidelines. *CPC* Cerebral Performance Categories scale, *GCS* Glasgow Coma Scale, *PCAS* Post-cardiac arrest syndrome, *PEEP* Positive end-expiratory pressure, *ROSC* Return of spontaneous circulation, *SpO*_*2*_ Peripheral oxygen saturation

The intervention is the inhalation of the blinded trial gas, which will be initiated upon admission to the intensive care unit (ICU) and will be continued for 18 h, along with TTM (Fig. 1). The H_2_ group will be ventilated with 2% H_2_ and titrated O_2_, whereas the control group will be ventilated with conventional titrated O_2_ through the gas inhalation system, as described below.

### Gas inhalation system

**Fig. 2:**
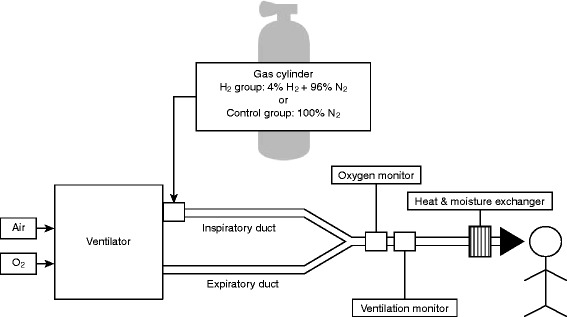
System of trial gas inhalation. The trial gas will be inhaled through this system. Trial gas will be supplied from a cylinder with constant flow, and it will be mixed with oxygen from the mechanical ventilator (SERVO-s®; MAQUET Critical Care AB) at the inspiratory duct. The cylinder is filled with 4% H_2_ and 96% nitrogen for the hydrogen group and 100% nitrogen for the control group. A tidal volume and fraction of inspired oxygen meter will be used during trial gas inhalation to monitor ventilation

The inhalation of the trial gas will be conducted using a previously described system [13]. The study gas will be supplied from a cylinder with constant flow and will be mixed with oxygen from the mechanical ventilator (SERVO-s®; MAQUET Critical Care AB, Solna, Sweden) at the inspiratory duct. The cylinder is filled with 4% H_2_ and 96% nitrogen for the HI group and with 100% nitrogen for the control group (Fig. 2). Thus, the maximum O_2_ concentration is limited to 50% with our current HI system because prefilled 4% H_2_ in N_2_ is mixed with oxygen to obtain 2% H_2_ containing O_2_. The optimum combination values of tidal volume, respiratory rate, fraction of inspired oxygen, and trial gas flow rate, which are common for both groups, have been predetermined to obtain the above-mentioned gas composition. In our HI system, there is a discrepancy in both volume and composition between the gas emitted from the ventilator and the gas inhaled by the patient. To overcome this phenomenon, we need to deactivate the alarm on the ventilator, which works as a fail-safe under ordinary circumstances. Alternatively, we will install extra devices to monitor the tidal volume and O_2_ concentration throughout the intervention to ensure safety.

### Mechanical ventilation

During the intervention, patients will be mechanically ventilated with mandatory volume-control ventilation according to the predefined combination. The oxidation target is peripheral oxygen saturation (SpO_2_) ≥ 94% or partial pressure of oxygen in arterial blood between 85 mmHg and 150 mmHg. The respiration target is a partial pressure of carbon dioxide in arterial blood between 35 mmHg and 45 mmHg. Patients with respiratory failure requiring > 50% O_2_, even with adequate PEEP to maintain sufficient oxygenation, or those experiencing SpO_2_ < 94% will be excluded from the trial. Patients will be sedated (propofol and/or midazolam), given analgesia (fentanyl), and paralyzed with neuromuscular blocking agents (rocuronium bromide) during the intervention. Following the completion of the intervention, the ventilator will be changed from the study gas inhalation system to the conventional ventilator available in the ICU. The ventilation mode will be tailored to each patient. Additionally, the neuromuscular blocking agents can be discontinued if they are unnecessary.

### Concurrent therapy

TTM will be performed in all patients. The body temperature will be controlled using surface or intravascular temperature management devices to achieve a target core temperature between 33 °C and 36 °C. The choice of the target temperature between 33 °C and 36 °C is determined by individual institutional policy. However, efforts will be made to achieve the target temperature as soon as possible, and the target temperature will be maintained for 24 h. Patients will be passively rewarmed to a core temperature of 36 °C over 48 h. Sedation will be stopped upon reaching a temperature of 36 °C in patients undergoing TTM at 32 °C to 35 °C. In patients who are managed with TTM at 36 °C, sedation will be stopped 72 h after initiation of the study gas inhalation. Hyperthermia will be avoided by using icepacks and nonsteroidal anti-inflammatory drugs to maintain a temperature of 37 ± 0.5 °C until 7 days post-ROSC for all patients.

Patients will be treated using standard therapies for cardiac diseases. Coronary angiography and percutaneous coronary interventions will be performed according to current guidelines and at the discretion of the treating physicians. Other diagnostic testing will be performed only as indicated after admission.

### Data collection and follow-up

**Fig. 3:**
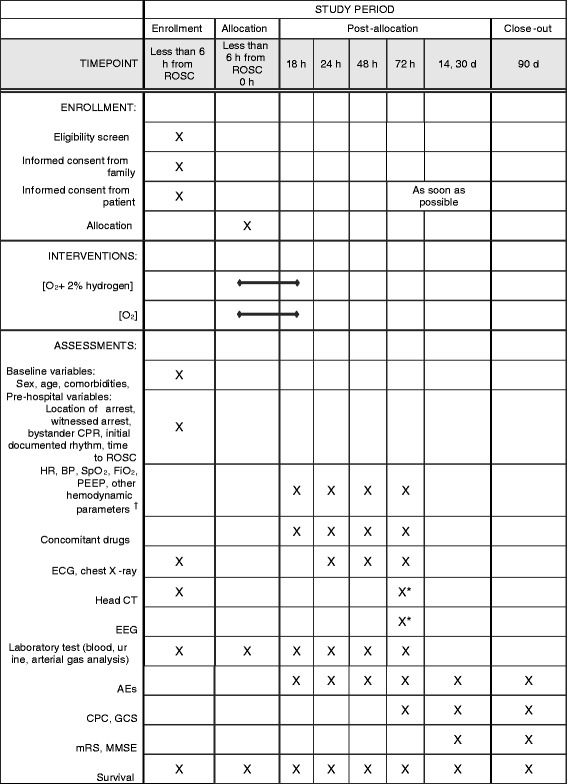
Standard Protocol Items: Recommendations for Interventional Trials (SPIRIT) figure depicting schedule of enrollment, interventions, and assessments. ^†^ Hemodynamic parameters will be obtained using commercially available systems (PiCCO®, MAQUET Critical Care AB; or EV1000®, Edwards Lifesciences, Irvine, CA, USA). * Perform head CT scan and EEG during the daytime after 72 h from the initiation of intervention. *ABG* Arterial blood gas analysis, *AEs* Adverse events, *BP* Blood pressure, *CAG* Coronary angiography, *CBC* Complete blood count, *CPC* Cerebral Performance Categories scale, *CT* Computed tomography, *ECG* Electrocardiogram, *EEG* Electroencephalogram, *ER* Emergency room, *FiO*_*2*_ Fraction of inspired oxygen, *GCS* Glasgow Coma Scale, *HR* Heart rate, *MMSE* Mini Mental State Examination, *mRS* Modified Rankin Scale, *PCI* Percutaneous coronary intervention, *PEEP* Positive end-expiratory pressure, *SpO*_*2*_ Peripheral oxygen saturation

Each participant’s baseline demographics and medical history will be collected. All patient data will be collected on a dedicated case report form (CRF). Vital signs, clinical biochemistry, physiological, and radiographic test results will be collected as scheduled (Fig. 3). Patients will be followed until 90 days after CA. Survival status will be recorded until 90 days after CA. The 90-day neurological evaluation will be performed by a neurologist or neurosurgeon at each institution and reported through the designated CRFs. Next, two prespecified neurologists outside the study group who are unaware of the treatment will determine the 90-day neurological outcome by evaluating the collected CRFs. Collected CRFs will be locked and stored at the Keio University School of Medicine. All patients in the study will be actively treated, and brain damage biomarkers will not be used for operational prognostication. All centers will be regularly monitored for source data documentation, and missing or questionable data will be completed and corrected by queries.

### Outcome measures

The primary outcome is the proportion of participants achieving a favorable 90-day neurological outcome as defined by CPCs of 1 and 2 [14]. Secondary outcomes include the 90-day survival rate, survival time, GCS score, modified Rankin Scale (mRS) score, and Mini Mental State Examination (MMSE) results. Cognitive impairment among OHCA survivors is well known, and MMSE is the most commonly used instrument for brief cognitive function screening [15, 16]. We will also collect GCS scores for supplementary evaluation of the prevalence of disturbance of consciousness.

### Adverse events

Patients with PCAS are already in a life-threatening condition following ischemia-reperfusion injury, and various clinical complications, including death, can manifest independent of HI. Therefore, persistent disability/incapacity or a life-threatening condition may not necessarily indicate a serious adverse event (SAE) if it was predictable from the patient’s clinical condition. No exceptional rules exist for defining or reporting adverse events (AEs) in studies on patients with PCAS. Therefore, in order to proceed with the study smoothly while fully considering safety, we need to define AEs and establish a reporting rule whereby any newly emergent conditions and clinically significant worsening of the patient’s underlying condition, including death, should be recorded in all circumstances. Thus, we defined an AE as any unfavorable medical occurrence that is temporally associated with the participant’s involvement in the research, regardless of whether it is considered to be related to participating in the study after HI initiation until the end of the 90-day follow-up period. A clinically unfavorable medical occurrence is defined as death, life-threatening worsening of conditions (Additional file 2), permanent or severe organ dysfunction (Additional file 2: Table S1), laboratory abnormalities (Additional file 2: Table S2), seizure, and any other condition that the investigators judge as representing a significant hazard. Considering the above-referenced characteristics of patients with PCAS, an SAE is defined as an AE that occurs within 72 h after the initiation of the intervention or death during the follow-up period. Safety variables will be recorded in a prespecified CRF.

### Sample size estimation

On the basis of published data [17], we expect the rate of favorable outcome achieved with the current comprehensive critical care, including TTM, in this population to be 50%. Because there are limited clinical data [13], we considered animal data [12] and assumed that the absolute risk reduction by HI is 15%; that is, the favorable neurological outcome rate improves from 50% to 65% with HI. A sample size of 167 patients in each group will provide 80% power to detect a 15% change in the proportion of good neurological outcomes (CPCs of 1 and 2), from 50% to 65%, with an overall significance level of 0.05 according to a two-sided χ^2^ test. A low rate of failure to follow-up is anticipated because of the short duration of the follow-up and the consideration of recent experiences in our previous study. Including an assumed incomplete patient data rate of 5%, 180 patients are required for each group, for a total sample size of 360.

### Statistical methods

All statistical analyses, including the interim analysis, will be thoroughly performed by independent statisticians who are not involved in patient treatment or outcome assessment. Statisticians are blinded to the allocation code. Statisticians will perform statistical analyses according to predetermined data handling and statistical methods, and there will be no arbitrary interference. All analyses will be performed on a full-analysis basis. Baseline characteristics by group will be compared using descriptive analyses. The proportion of patients achieving good neurological outcomes at the end of the follow-up will be compared using the Pearson χ^2^ test, which will be the primary result of the trial. With regard to the secondary outcomes, the 90-day survival rate will be compared using the Pearson χ^2^ test; the duration of survival will be analyzed using Cox regression analysis; and other neurological outcomes, such as mRS, GCS, and MMSE scores, will be compared using the Mann-Whitney *U* test. All tests will be two-tailed, and a *P* value of 0.05 will be considered statistically significant. All data analyses will be performed using SAS version 9.3 software (SAS Institute, Cary, NC, USA).

### Interim analysis

The interim analysis of safety will be conducted by the data and safety monitoring committee (DSMC) to determine whether to continue the study after the first 100 patients have been followed for 90 days. This analysis will be performed only for death (within the 90th day of the study). In the interim analysis, we will make a statistical estimate comparing mortality between the hydrogen and control groups. For each group, an independent beta distribution [β(1,1)] will be used as a prior distribution, and the posterior distribution of the mortality rate of each group will be estimated on the basis of number of deaths observed. Based on the aforementioned distribution, the criteria for the discontinuation of the study will be met when the probability that the mortality rate of the hydrogen group is higher than that of the control group is ≥ 0.9875.

### Data and safety monitoring committee

The DSMC is an independent group consisting of clinicians who have experience in the management of ICU patients and a statistician who has experience in the execution, monitoring, and analysis of clinical trials. DSMC membership is restricted to nonparticipating clinicians without conflicts of interest. The DSMC will be responsible for safeguarding the interests of trial participants, assessing the causal relationship between reported AEs and the intervention, and the interim analysis. The DSMC will provide recommendations regarding stopping or continuing the trial to the principal investigator of the trial.

### Auditing

Auditing will be outsourced to a contract research organization independent of the investigators and the sponsor. Four institutions will be randomly chosen annually, and auditing will be performed according to the predefined procedure.

## Discussion

HI is a promising treatment option for PCAS. Because PCAS is observed in a unique group of patients and has a serious and urgent nature, it is rarely studied in double-blind RCTs. The HYBRID II trial will be the first randomized double-blind trial to evaluate the efficacy of HI.

Animal experimental data support the benefits of HI for PCAS [11, 12]. Therapeutic effects of HI at concentrations ranging from 1.3% to 3% have been reported in various models. We have chosen an H_2_ concentration of 2% because the protective effect against acute oxidative stress plateaued at over 2% in previous reports [8, 18]. Synergistic effects of TTM and HI have been reported in rodent PCAS models [11, 12]. TTM and HI may share the same theoretical neuroprotective mechanisms. However, other yet to be defined mechanisms are speculated to exist.

Although H_2_ gas is flammable, concentrations < 4% together with oxygen at room temperature are incombustible. As indicated by the second law of thermodynamics, although several physical processes that satisfy the first law are possible, the only processes that occur in nature are those for which the entropy of the system either remains constant or increases. Thus, exhaled H_2_ diffuses instantly, not accumulating or resulting in an increased concentration exceeding that of the inspiratory H_2_ concentration. Therefore, 2% H_2_ gas can be solicitously administered in a hospital.

HI at a therapeutic dose has been reported to have no adverse effects on vital signs [18]. No unfavorable effects have been reported with the prolonged repetitive inhalation of H_2_ to prevent decompression sickness in healthy deep-sea divers [19, 20].

Comatose post-CA patients generally require mechanical ventilation. Therefore, H_2_ must be administered through a mechanical ventilator circuit. We have successfully devised a ventilator system that enables simultaneous administration of titrated H_2_ and O_2_ (Fig. 2) using a clinically available ventilator. A significant point to emphasize is that platinum is a catalyst for the oxidation reaction of H_2_. Thus, even if H_2_ itself is incombustible under 4% concentration at room temperature, the platinum surface may be overheated in the presence of H_2_ and O_2_, eventually leading to mechanical failure. This is essential because most of the current clinically available ventilators are equipped with a platinum hot manometer as a flow sensor, thereby limiting ventilator options for the administration of H_2_ gas. We chose an existing ventilator equipped with an ultrasonic flow sensor (Servo-s®). The feasibility and safety of HI using this system was confirmed in a small sample size of subjects with PCAS [13]. A clinical trial evaluating the efficacy of HI for PCAS is eagerly anticipated [21].

Our HI system has several limitations. First, the maximum O_2_ concentration is limited to 50%. Although PEEP will be tailored to maintain sufficient oxygenation, patients requiring > 50% O_2_ must be excluded. Second, HI is limited to 18 h because of space limitations for the installation of cylinders in the Japanese ICU. The gas cylinder that will be used in this trial is capable of providing gas for approximately 10 to 12 h, depending on the flow rate. Considering the space limitation, two cylinders per patient is the maximum. Thus, the trial gas inhalation duration was set for 18 h. There are no conclusive studies on the optimal duration of HI after CA, even in animal models; thus, 18 h might be short for some patients. Third, a time lag between ROSC and HI initiation is inevitable because the HI system will need to be installed in the ICU. H_2_ has been reported to ameliorate ischemia-reperfusion injury in part by radical-scavenging effects; it is easily assumed that earlier initiation of HI after ROSC in the ER may demonstrate better results. However, our results derived from animal experiments revealed that the benefit of HI is similar, even when it is initiated following ROSC [12]. The time delay for obtaining consent is minimal because consent is usually obtained from a proxy while the patient is sent for emergent revascularization. A deferred consent approach was not chosen, because consent from a proxy was mandated by the Japanese government for approval of this trial as an advanced medicine clinical trial. Based on the abovementioned reasons, a time lag < 6 h between starting HI after ROSC is considered realistic. According to our previous experience, it is expected that the refusal rate will not be sufficient to produce a selection bias.

We have designed a pragmatic randomized trial with wide inclusion criteria after taking previous trials, clinical guidelines, and current clinical practices into consideration. Wide inclusion criteria will strengthen the generalizability of our results.

In this trial, we will arrange randomization and blinding in a unique way by using ingredient-concealed, prerandomized cylinders. It is practically impossible to stock certain sets of large cylinders at each hospital and choose them according to central computer-based randomization. The blinding and 1:1 allocation will be maintained without confusion by simply using the delivered cylinders in numerical order at each site. Scrupulous trial design, including monitoring and auditing by external contract research organizations, is planned to maintain compliance and eventually to obtain high-quality evidence. Moreover, this trial has been approved as an advanced medicine clinical trial whose aim is to obtain future regulatory approval by the Ministry of Health, Labor and Welfare of the Japanese government.

H_2_ is one of the most abundant substances in the universe, and it is far less expensive and is effective at lower concentrations than other noble gases that have been studied for PCAS [22]. The unique features of HI include a less invasive nature, easy and safe administration, and no obvious known adverse effects. Therefore, our expectation is that once H_2_ is demonstrated to be effective, HI will have the potential to be widely applied to patients with PCAS.

### Trial status and time line

As of 19 October 2017, 2 patients were enrolled and recruitment is ongoing. Approximately 15 institutions are preparing to begin patient enrollment. A total of 360 patients will be recruited for the trial within 3 years.

## Additional files

Additional file 1: Completed Standard Protocol Items: Recommendations for Interventional Trials (SPIRIT) checklist of recommended items to address in a clinical trial protocol. (DOC 125 kb)

Additional file 2: Definition of adverse event. (DOCX 26 kb)

## Abbreviations

ABG: Arterial blood gas analysis
AE: Adverse event
BP: Blood pressure
CA: Cardiac arrest
CAG: Coronary angiography
CBC: Complete blood count
CPC: Cerebral Performance Categories scale
CRF: Case report form
CT: Computed tomography
DSMC: Data and safety monitoring committee
ECG: Electrocardiogram
EEG: Electroencephalogram
ER: Emergency room
FiO_2_: Fraction of inspired oxygen
GCS: Glasgow Coma Scale
H_2_: Molecular hydrogen
HI: Hydrogen gas inhalation
HR: Heart rate
HYBRID II: Efficacy of inhaled HYdrogen on neurological outcome following BRain Ischemia During post-cardiac arrest care
ICU: Intensive care unit
ILCOR: International Liaison Committee on Resuscitation
MMSE: Mini Mental State Examination
mRS: Modified Rankin Scale
OHCA: Out-of-hospital cardiac arrest
PCAS: Post-cardiac arrest syndrome
PCI: Percutaneous coronary intervention
PEEP: Positive end-expiratory pressure
RCT: Randomized, double-blind, placebo-controlled trial
ROSC: Return of spontaneous circulation
SAE: Serious adverse event
SPIRIT: Standard Protocol Items: Recommendations for Interventional Trials
SpO_2_: Peripheral oxygen saturation
TTM: Targeted temperature management
UMIN: University Hospital Medical Information Network
