# Supplementary material for: Efficacy of inhaled HYdrogen on neurological outcome following BRain Ischemia During post-cardiac arrest care (HYBRID II trial): study protocol for a randomized controlled trial
Source: Trials. 2017 Oct 23;18:488. doi: 10.1186/s13063-017-2246-3 (PMC5651618; doi:10.1186/s13063-017-2246-3)
Supplement: Supplementary file 2 — Definition of adverse event. (DOCX 26 kb) [file 13063_2017_2246_MOESM2_ESM.docx]

**Additional file 1**

Tamura T^*^, Hayashida K, Sano M, et al. Efficacy of inhaled HYdrogen on neurological outcome following BRain Ischemia During post-cardiac arrest care (HYBRID II trial): study protocol for a randomized controlled trial

**Clinical trial identifier:**

UMIN000019820, Hydrogen Inhalation Therapy for Patients with Post Cardiac Arrest Syndrome (Phase II, multicenter, prospective, randomized, double-blind, placebo-controlled trial)

***Adverse Event***

Organ failure can occur in almost every organ due to severe whole body ischemic-reperfusion injury and underlying disease in post-cardiac arrest syndrome (PCAS). We therefore adopted the Sequential Organ Failure Assessment (SOFA) score, a well-established and commonly used score in the critical care setting for the assessment of organ failure, to define life threatening conditions and severe organ dysfunction for adverse event (AE) reporting. It has previously been reported that the mortality rate is 50% in patients with SOFA scores >11, including for the central nervous system.^1^ The score for the central nervous system in the SOFA score for GCS less than 8 is 3. Therefore, we defined a life-threatening condition as a SOFA score >8 without a score for the central nervous system, and a score change of more than +2 in each variable of the SOFA score is considered severe organ dysfunction. A change in score of +1 in each variable occurs frequently in patients with PCAS, and those are usually reversible. Thus, by defining a score change of +2 as an occurrence of organ dysfunction, a minor change in a near-threshold value will not be falsely recognized as organ dysfunction.

| **Variables** | **SOFA Score** | | | | |
| --- | --- | --- | --- | --- | --- |
|  | 0 | 1 | 2 | 3 | 4 |
| **Respiratory**  PaO_2_/FiO_2_, mm Hg | > 400 | ≤ 400 | ≦300 | ≦200  with respiratory support | ≦100  with respiratory support |
| **Coagulation**  Platelets, ×10^3^/μL | > 150 | ≤ 150 | ≦100 | ≦50 | ≦20 |
| **Liver**  Bilirubin, mg/dL | < 1.2 | 1.2-1.9 | 2.0-5.9 | 6.0-11.9 | >12.0 |
| **Cardiovascular**  Hypotension | No hypotension | MAP < 70 mmHg | dopamine ≦5  or  dobutamine（any doses） | dopamine >5,  epinephrine ≦0.1,  or  norepinephrine ≦0.1 | dopamine ＞15,  epinephrine ＞0.1,  or  norepinephrine ＞0.1 |
| **Renal**  Creatinine, mg/dL | < 1.2 | 1.2 - 1.9 | 2.0 - 3.4 | 3.5 - 4.9 | >5.0 |

Additional file 1: Table S1. Sequential Organ Failure Assessment Score

MAP = mean arterial pressure. Catecholamine doses are given as μg/kg/minute for at least 1 hour.

We adopted both relative and absolute assessment to judge whether a laboratory test abnormality is an AE or not. In a relative assessment, the value is compared with that obtained in the ER. Values fulfilling both relative and absolute assessment will be considered AEs. The condition of each variable is as shown in Supplementary Table 2.

Additional file 1: Table S2. Laboratory test variables and thresholds

| Variables | Units | Relative assessment | | Absolute assessment |
| --- | --- | --- | --- | --- |
| **Complete Blood Count** |  |  |  | |
| WBC | /μL | > ×2 | > 20,000 | |
|  |  | < ×0.5 | < 3,000 | |
| Hemoglobin | g/dL | < ×0.5 | < 7 | |
| Platelets | /μL | > ×1.5 | > 450,000 | |
|  |  | < ×0.33 | < 80,000 | |
| **Coagulation^*^** |  |  |  | |
| aPTT | second | > ×2 | > 100 | |
| PT (INR) | INR | > ×2 | > 2.5 | |
| FDP | μg/mL | > ×5 | > 40 | |
| **Chemistry** |  |  |  | |
| CK^†^ | IU/L | > ×3 | > 800 | |
| AST^†^ | IU/L | > ×3 | > 100 | |
| ALT | IU/L | > ×3 | > 100 | |
| ALP | IU/L | > ×3 | > 800 | |
| LDH^†^ | IU/L | > ×3 | > 800 | |
| TP | g/dL | < ×0.67 | < 4.5 | |
| TB | mg/dL | > ×3 | > 5.0 | |
| UN | mg/dL | > ×2 | > 50 | |
| Cr | mg/dL | > ×2 | > 3.0 | |
| UA | mg/dL | > ×1.5 | > 10 | |
| CRP | mg/dL | > ×5 | > 10 | |

*Value changes in coagulation variables due to the use of anticoagulants will not be considered meaningful．†Changes in CK, AST, and LDH will not be considered significant in participants after acute myocardial infarction.

***Reference***

1. Ely EW, Laterre PF, Angus DC, Helterbrand JD, Levy H, Dhainaut JF, et al. Drotrecogin alfa (activated) administration across clinically important subgroups of patients with severe sepsis. Crit Care Med 2003;31(1):12-9.
